# Supplementary material for: Association between benign prostatic hyperplasia and suicide in South Korea: A nationwide retrospective cohort study
Source: PLoS One. 2022 Mar 10;17(3):e0265060. doi: 10.1371/journal.pone.0265060 (PMC8912228; doi:10.1371/journal.pone.0265060)
Supplement: S1 Table — (DOC) [file pone.0265060.s001.doc]

**Supplementary Table 1. Hazard ratios (95% CI) for suicide among patients with benign** prostatic hyperplasia according to different income levels

| **Characteristics** | **Suicide rates (95% CI)** | **Multivariable adjusted HR (95% CI)** | ***p* for interaction** |
| --- | --- | --- | --- |
| Medical aid |  |  | 0.59 |
| Without benign prostatic hyperplasia | 123.3 (95.8 to 156.5) | 1.00 (reference) |
| With benign prostatic hyperplasia | 201.3 (114.6 to 329.8) | 1.62 (0.87 to 3.02) |
| Low income level |  |  |
| Without benign prostatic hyperplasia | 76.6 (68.4 to 85.7) | 1.00 (reference) |
| With benign prostatic hyperplasia | 102.9 (74.5 to 138.8) | 1.30 (0.91 to 1.86) |
| Middle income level |  |  |
| Without benign prostatic hyperplasia | 56.3 (50.4 to 62.8) | 1.00 (reference) |
| With benign prostatic hyperplasia | 103.4 (78.6 to 133.5) | 1.52 (1.11 to 2.08) |
| High income level |  |  |
| Without benign prostatic hyperplasia | 45.8 (39.4 to 52.8) | 1.00 (reference) |
| With benign prostatic hyperplasia | 72.5 (51.8 to 98.8) | 1.59 (1.09 to 2.32) |

Suicide rates are expressed as incidence density per 100,000 person-years

Multivariable adjusted model was adjusted for age, geographical location, comorbidities, disabilities, mental health, and income level

*P* for interaction was tested using likelihood ratio test
