# Supplementary material for: Association between benign prostatic hyperplasia and suicide in South Korea: A nationwide retrospective cohort study
Source: PLoS One. 2022 Mar 10;17(3):e0265060. doi: 10.1371/journal.pone.0265060 (PMC8912228; doi:10.1371/journal.pone.0265060)
Supplement: S2 Table — (DOC) [file pone.0265060.s002.doc]

**Supplementary Table 2. Hazard ratios (95% CI) for suicide among patients with benign prostatic hyperplasia according to presence of mental disorders**

| **Mental health disorder** | **Suicide rates (95% CI)** | **Multivariable adjusted HR (95% CI)** | ***p* for interaction** |
| --- | --- | --- | --- |
| No mental health disorder |  |  | 0.75 |
| Without benign prostatic hyperplasia | 49.8 (46.0 to 53.8) | 1.00 (reference) |
| With benign prostatic hyperplasia | 64.4 (50.8 to 80.5) | 1.36 (1.05 to 1.76) |
| People with mental disorder |  |  |
| Without benign prostatic hyperplasia | 162.8 (143.0 to 184.5) | 1.00 (reference) |
| With benign prostatic hyperplasia | 199.0 (157.1 to 248.7) | 1.66 (1.24 to 2.21) |

Suicide rates are expressed as incidence density per 100,000 person-years

Multivariable adjusted model was adjusted for age, geographical location, comorbidities, disabilities, mental health, and income level

*P* for interaction was tested using likelihood ratio test
