# Supplementary material for: Association between benign prostatic hyperplasia and suicide in South Korea: A nationwide retrospective cohort study
Source: PLoS One. 2022 Mar 10;17(3):e0265060. doi: 10.1371/journal.pone.0265060 (PMC8912228; doi:10.1371/journal.pone.0265060)
Supplement: Attachment — Submitted filename: Response to the Reviewers Comments (220131).doc [file pone.0265060.s004.doc]

**Manuscript ID: PONE-D-21-21842.R1**

**Original title:**

**Association between Benign Prostatic Hyperplasia and Suicide in South Korea: a Nationwide Retrospective Cohort Study**

**To the Editors and Reviewers**:

We thank you for careful reading of the manuscript and helpful comments and suggestions from reviewers. We are submitting reviewers’ comments together, and plan how we revised our paper according to the reviewers’ suggestions and comments. Changes made to the text are marked with highlights changes and track change mode in the revised manuscript.

**Comments from Academic Editor:**

1. Can you add explanation about what the reviewer considers a limitation of the generalisability of the findings?

**Answer:** In my opinion, reviewer is pointing out that we have to consider the specific situation of South Korea – South Korea has the highest suicide rate among OECD country, although South Korea has achieved remarkable success in combining rapid economic growth with significant poverty reduction. There are unique cultural and social background behind this highest suicide rate in South Korea [1-2].

In addition, there is a cultural barrier that are reluctant to visit psychiatry for mental disorder. In particular, there were severe stigma and prejudice toward patients with mental disorders such as depression [3]. In addition, physical illness is common cause for suicide among elderly people [4].

Considering these points, depression due to BPH may be relatively undiagnosed or untreated, and can easily lead to suicide compared to the other countries. These cultural differences (stigma, cultural barrier to visit psychiatry) can lead to difficulties in generalization of our study findings to the situation of other country.

1. Lee SU, Park JI, Lee S, Oh IH, Choi JM, Oh CM. Changing trends in suicide rates in South Korea from 1993 to 2016: a descriptive study. BMJ Open. 2018;8(9):e023144.

2. Hong J, Knapp M. Impact of macro-level socio-economic factors on rising suicide rates in South Korea: panel-data analysis in East Asia. J Ment Health Policy Econ. 2014;17(4):151-62.

3. Park J, Jeon M. The stigma of mental illness in Korea. Journal of Korean Neuropsychiatric Association. 2016;55(4):299-309.

4. https://www.statista.com/statistics/1230755/south-korea-number-of-suicides-by-reason/

**Comments from Reviewer #3:**

1. South Korea has one of the world's highest suicide rates per capita. The reasons for this are not clear, but are likely to be multifactorial. As such, the findings in the authors' manuscript may not be generalizable to a different country with lower per capita suicide rates. I would add this as a limitation.

**Answer:** Thank you for comments. We fully agree with the reviewer's opinion that our study finding are generally applicable to other countries. Therefore, the following sentence has been added to the limitation part of the discussion:

(Page 16, Line 326- Page 17, Line 331) Finally, our study findings cannot be easily generalized to other countries. South Korea is considered as a developed country with a very rapid economic growth. At the same time, South Korea has the highest suicide rate among OECD countries. While South Korea has the advantage of physically and economically easy access to medical service, there are cultural barriers that are reluctant to visit psychiatry for mental disorder.
