# Supplementary material for: Association between benign prostatic hyperplasia and suicide in South Korea: A nationwide retrospective cohort study
Source: PLoS One. 2022 Mar 10;17(3):e0265060. doi: 10.1371/journal.pone.0265060 (PMC8912228; doi:10.1371/journal.pone.0265060)
Supplement: Attachment — Submitted filename: Response to the Reviewers Comments (220221).doc [file pone.0265060.s005.doc]

**Manuscript ID: PONE-D-21-21842.R2**

**Original title:**

**Association between Benign Prostatic Hyperplasia and Suicide in South Korea: a Nationwide Retrospective Cohort Study**

**To the Editors and Reviewers**:

We thank you for careful reading of the manuscript and helpful comments and suggestions from reviewers. We are submitting reviewers’ comments together, and plan how we revised our paper according to the reviewers’ suggestions and comments. Changes made to the text are marked with highlights changes and track change mode in the revised manuscript.

**Comments from Academic Editor:**

1. Can you please add the (new) reference(s), that you provided in your answer regarding the limitations for generalizability, in the text of the manuscript as well?

**Answer:** As the editor suggested, we have added new references (no.33- no.34) to the limitation part of external generalization as follows:

(Page 16, Line 326 – Page 17, Line 2) Finally, our study findings cannot be easily generalized to other countries. South Korea is considered as a developed country with a very rapid economic growth [33]. At the same time, South Korea has the highest suicide rate among OECD countries [15]. While South Korea has the advantage of physically and economically easy access to medical service, there are cultural barriers that are reluctant to visit psychiatry for mental disorder [34].

References)

33. Hong J, Knapp M. Impact of macro-level socio-economic factors on rising suicide rates in South Korea: panel-data analysis in East Asia. J Ment Health Policy Econ. 2014;17:151-162.

34. Park JI, Jeon M. The Stigma of Mental Illness in Korea. J Korean Neuropsychiatr Assoc. 2016:55:299-309.

Paper of Hong J et al (No.33) pointed out that suicide rate in South Korea especially higher compared to other Asian countries, despite of similarities in geography and culture. The findings highlight the differential associations between social changes and suicide rates at various stages over a person's life course.

Paper of Park JI (No.34) discussed the stigma of psychiatric disorders in Korea and the reluctance to visit a psychiatric clinic/hospital.

2. Please review your reference list to ensure that it is complete and correct. If you have cited papers that have been retracted, please include the rationale for doing so in the manuscript text, or remove these references and replace them with relevant current references. Any changes to the reference list should be mentioned in the rebuttal letter that accompanies your revised manuscript. If you need to cite a retracted article, indicate the article’s retracted status in the References list and also include a citation and full reference for the retraction notice.

**Answer:** We checked all references and confirmed that they are correct.
